# The Usefulness of Immunohistochemistry in the Differential Diagnosis of Lesions Originating from the Myometrium

**DOI:** 10.3390/ijms20051136

**Published:** 2019-03-06

**Authors:** Piotr Rubisz, Michał Ciebiera, Lidia Hirnle, Magdalena Zgliczyńska, Tomasz Łoziński, Piotr Dzięgiel, Christopher Kobierzycki

**Affiliations:** 1 First Department of Gynecology and Obstetrics, Wroclaw Medical University, 50-368 Wrocław, Poland; piotr.rubisz@gmail.com (P.R.); lidia.hirnle@umed.wroc.pl (L.H.); 2 Second Department of Obstetrics and Gynecology, The Center of Postgraduate Medical Education, 01-809 Warsaw, Poland; 3 Students’ Scientific Association at the I Department of Obstetrics and Gynecology, Medical University of Warsaw, 02-015 Warsaw, Poland; zgliczynska.magda@gmail.com; 4 Department of Obstetrics and Gynecology Pro-Familia Hospital, 35-001 Rzeszów, Poland; tomasz.lozinski@pro-familia.pl; 5 Division of Histology and Embryology, Department of Human Morphology and Embryology, Wroclaw Medical University, 50-368 Wroclaw, Poland; piotr.dziegiel@umed.wroc.pl (P.D.); ch.kobierzycki@gmail.com (C.K.); 6 Department of Physiotherapy, University School of Physical Education, 51-612 Wroclaw, Poland

**Keywords:** uterine fibroid, leiomyoma, smooth muscle tumor of uncertain malignant potential, leiomyosarcoma, myometrium, immunohistochemistry, marker, pathology, tumor, diagnosis

## Abstract

Uterine leiomyomas (LMs), currently the most common gynecological complaint around the world, are a serious medical, social and economic problem. Accurate diagnosis is the necessary prerequisite of the diagnostic-therapeutic process. Statistically, mistakes may occur more often in case of disease entities with high prevalence rates. Histopathology, based on increasingly advanced immunohistochemistry methods, is routinely used in the diagnosis of neoplastic diseases. Markers of the highest sensitivity and specificity profiles are used in the process. As far as LMs are concerned, the crux of the matter is to identify patients with seemingly benign lesions which turn out to be suspicious (e.g., atypical LM) or malignant (e.g., leiomyosarcoma (LMS)), which is not uncommon. In this study, we present the current state of knowledge about the use of immunohistochemical markers in the differential diagnosis of LM, atypical LM, smooth muscle tumors of uncertain malignant potential (STUMP), and LMS, as well as their clinical predictive value.

## 1. Introduction

### 1.1. Epidemiology and Etiopathology of Myometrial Neoplasms

Tumors arising from the smooth muscle cells of the uterus are the most common neoplasms of the female genital tract around the world [1,2], chief among them leiomyomas (LMs), which are benign lesions of the uterus. Their prevalence is age-dependent, reaching 70–80% among women >50 years of age, and has been estimated at approximately 40–60% among women <35 years of age, depending on the population [3,4]. The results of the population observations have been confirmed by hysterectomy preparations—LMs are diagnosed in 77–96% of the patients [5,6], followed by lipoleiomyomas, which are relatively common LM variants (approx. 2.3%) among postmenopausal women who require surgical intervention [6].

Despite the fact that LMs are very common, the etiopathogenesis and pathophysiology of these benign tumors remain unclear [7,8,9,10]. The risk factors for the development of LMs include modifiable factors [1], e.g., dietary components [7,11,12], and non-modifiable factors, e.g., genetic [13,14], both in case of spontaneous changes [15] as well as autosomal dominant heredity [16].

According to the available literature, LM growth depends mostly on steroid hormones [13,17,18,19]. Nowadays, progesterone is believed to be the dominant factor in LM pathophysiology and growth stimulation [13,19,20]. The effect of progesterone on the development of uterine fibroids consists in the overexpression of cytokine-related genes and the increase of selected growth factor concentrations, e.g., transforming growth factors (TGFs), vascular endothelial growth factor (VEGF), tumor necrosis factor-α (TNF-α), and many others [7,8,9,21]. LMs are benign lesions with favorable prognosis but, due to their prevalence and considerable similarities to malignant tumors which are also derived from the body of the uterus, detailed histopathological evaluation is necessary to exclude dormant leiomyosarcomas and to determine their potential for malignant transformation [22]. According to the available literature, leiomyosarcomas (LMSs) account for 1–2% of all malignant tumors of the uterus [22], and remain the most common sarcomas of the female reproductive tract [22]. Approximately 1 in 100,000 people are diagnosed with LMSs [23]. According to Kho et. al., occult uterine sarcoma occurs in 0.089% of the cases or 1 in 1124 hysterectomies in women undergoing surgery for benign gynecologic indications [24], while Graebe et al., reported an even higher risk –0.22% [25]. Zhao et al. (2015), calculated the risk for unexpected uterine sarcomas at 0.47% [26].

### 1.2. Differentiation and Advanced Pathological Diagnostics in Myometrial Neoplasm

Determination of the hormone receptor status of the tumor cells allows the prognosis and assessment of the dynamics of the neoplastic process [22,27]. In the case of LMs, identification of patients with a high risk for malignant transformation or dynamic tumor growth may help to decide on the treatment plan and/or the extent of the operative treatment, or pharmacotherapy for non-surgical management [27,28,29,30]. It is a well-known fact that numerous regulatory mechanisms of proliferation are disturbed in cancer cells, as is the case with LM, whose growth becomes a self-stimulatory, often tumor volume-dependent process [7,8,13].

Several well-described diagnostic markers are used in the diagnosis of neoplastic lesions, chief among them Ki-67 antigen, p53 protein and steroid receptors: estrogen (ER) and progesterone (PR) [22,31]. These markers are used to assess the biology of the myometrial lesions and for differential diagnosis of tumors with atypical presentation. Nevertheless, histopathological evaluation sometimes remains inconclusive, which is reason enough to continue the search for more reliable markers. Apart from the aforementioned markers, the following, whose diagnostic applicability is yet to be determined, are currently being tested: 

(a) proteins that control the cell cycle and have a direct influence on the dynamics of proliferation, e.g., p16 [32,33,34]; cyclin D1 [35,36]; Bcl-2 [37]; proliferating cell nuclear antigen (PCNA) [38];

(b) TGFs [7]; epidermal growth factor (EGF) [39]; VEGF [40]; insulin-like growth factors (IGFs) [41];

(c) proteins which are responsible for intercellular interactions: mucins [42]; galectins [39,42];

(d) proteins which are responsible for muscle cell contraction: caldesmon [43,44]; calponin [36,44]; α-smooth muscle actin [9,45];

(e) cytoskeletal proteins: vimentin, desmin, nestin, keratin [46,47].

### 1.3. Classification of Uterine Smooth Muscle Tumors

The differentiation between benign and malignant myometrial lesions continues to present a considerable challenge for pathologists, and sometimes the final diagnosis remains inconclusive [22,48]. A number of criteria have been suggested in an attempt to unify the classification of the uterine tumors [22,48,49,50]. Based on tumor morphology and biology, four groups of myometrial tumors have been distinguished [51]. The current classification of smooth muscle neoplasms is presented in Table 1 [51].

At present, a number of criteria are used for the differentiation and the final diagnosis [22,49]. Topographic hematoxylin-eosin (HE) staining is commonly used in anatomic pathology diagnostics to evaluate tissue architecture [22,52]. The criteria presented in Table 2 [53,54] or the Stanford University Criteria presented in Table 3 [55], are applicable for the differentiation between benign and malignant lesions.

Among malignant myometrial tumors, LMSs comprise approximately one-third of all uterine sarcomas. The annual incidence rate for LMSs has been calculated at 0.7–1 in 100,000 women [23,56]. LMSs are a relatively rare occurrence but have high malignancy potential and poor prognosis. The 5-year survival rate for LMSs is 50% in cases without metastases [57]. The presence of distant metastases significantly worsens the already unfavorable prognosis. According to most clinical studies, progression-free survival for patients with distant metastases is 12–15 months [58].

In light of the above, accurate diagnosis and pre-operative histopathological evaluation are essential [29,59]. Nevertheless, we still lack tools to distinguish categorically between benign and malignant lesions. It seems that possible disproportions in the concentrations of selected growth factors among patients with LMSs as compared to LMs might be a turning point as far as improved pre-operative detection of LMS is concerned. However, this subject has been discussed elsewhere [60].

The existence of lesions of uncertain potential, which further complicates the diagnostic process, presents a challenge for both pathologists and clinicians and is cause for concern to the affected patient. It is described as smooth muscle tumor of uncertain malignant potential (STUMP) according to the current classification of myometrial lesions. Tumors from that group have diverse structure and unclear clinical course [48,61,62]. To the best of our knowledge, there have been no reports or observational studies about patients with STUMP after surgical interventions [63]. Due to considerable clinical and morphological similarities between atypical, STUMP, and LMs lesions, advanced histopathological differentiation is necessary [62,64], and only a diagnostic process that includes immunohistochemical (IHC) evaluation may offer that.

Tumor differentiation is additionally complicated by the fact that lesions not derived from the myometrial cells may still arise in the myometrium. Based on the histological structure of a uterus, which consists of endo-, myo- and perimetrium, we know that the endometrium is composed of the epithelium and lamina propria. The latter is a connective tissue of mesenchymal origin as well as the elements of the myometrium. It is vital to bear that in mind during histopathological diagnosis and differentiation between myometrial lesions and tumors such as endometrial stromal sarcoma. The available literature reports suggest using diagnostic panels for the differentiation of the aforementioned lesions [36]. 

Due to the complexity of differentiating between myometrial changes, physicians and diagnosticians often find differentiation and the process of decision-making about further management challenging. The aim of our study was to systematize the knowledge on the currently used IHC markers and other markers, whose diagnostic potential remains to be fully validated. Also, the up-to-date opinions about the clinical practice for differential diagnosis are presented.

## 2. Methodology of Obtaining Data and Data Analysis

We conducted a search in PubMed of the National Library of Medicine and Google Scholar. Databases were extensively searched for all original and review articles/book chapters published in English until September 2018 and related to myometrial neoplasms using the following keywords (one or in combinations): uterine fibroid; uterine leiomyoma; uterine leiomyosarcoma; uterine sarcoma; smooth muscle tumor of uncertain malignant potential (STUMP); immunohistochemistry (IHC). Moreover, additional articles from the reference sections of the reviewed articles were searched. Overall, most relevant articles were reviewed and included as appropriate.

## 3. Available Immunohistochemical Markers

As mentioned above, tumors arising from the smooth muscle cells of the uterus may present a considerable challenge for pathomorphologists. IHC, owing to its steady development and advances, has become one of the main diagnostic tools in anatomic pathology in general. As far as histopathological evaluation of myometrial tumors is concerned, experts agree that no therapeutic intervention should be initiated without additional IHC testing, regardless of the primary diagnosis. Lack of compliance with this recommendation might result in false positive or false negative results and expose the affected patient to the risks associated with a failure to detect a malignant lesion [22,27,65].

IHC markers which may be useful in differentiating between benign and malignant myometrial tumors are presented below. Additionally, marker-dependent implications for clinical management and prognosis for the patients have been discussed. 

### 3.1. Markers with Strong Evidence 

#### 3.1.1. Ki-67

The Ki-67 proliferation antigen is the most common IHC marker in laboratory practice [22,31]. Despite being first described by Gerdes et al. as early as 1983, it continues to be the gold standard for the evaluation of the intensity of proliferative processes, and its expression in various neoplastic diseases has prognostic and predictive value [66]. The Ki-67 protein is expressed only in the proliferative phase of a cell (late G1, S, G2, and mitotic phases). Ki-67 antibody (MIB-1), used in the diagnostic process, makes IHC analysis more repeatable and accurate [67]. IHC testing confirmed that Ki-67 expression as a cellular marker for proliferation increases with tumor aggressiveness. Mayerhofer et al. (2004), demonstrated a significantly higher Ki-67 expression in the case of LMS vs. STUMP (*p =* 0.0001) and LMS vs. LM (*p* = 0.0002). At the same time, these authors found no statistically significant differences for STUMP vs. LM (*p* = 0.491) [68]. Mittal and Demopoulos (2001) reported similar results for LMS vs. STUMP and LMS vs. LM, and claimed that Ki-67 may be applicable in differentiating between STUMP vs. LM. Ki-67 expression level exceeded 15% in 11 out of 12 cases of LMS. Expression at the level of 5–10% was observed in 6 out of 7 STUMP cases. Importantly for result interpretation, Ki-67 expression was present in only 1 out of 15 cases of cellular LM, which is consistent with the biology of slow-growing benign lesions [69]. Petrović et al. (2010), found no Ki-67 expression in LM (LMS vs. LM (*p* = 0.0001) and STUMP vs. LM (*p* = 0.0001)), which indicated high diagnostic value of the marker in question. In their study, LMS vs. STUMP did not reach the level of statistical significance [70]. In 2009, Lee et al., reported that Ki-67, both as an isolated marker and in combination with p16 and p53, demonstrated a 92% sensitivity and a 98% specificity in differentiating between LMS and LM (65% LMS; 0% LM > 10% Ki-67 proliferation index *p* < 0.001) [71]. 

The literature offers reports about a positive correlation between Ki-67 expression and tumor aggressiveness, as well as clinical advancement of the disease in the case of LMS. Akhan et al. (2005), observed prolonged survival among patients with low Ki-67 expression (*p* = 0.034) [72], which is consistent with the findings of Mayerhofer et al. (2004), who demonstrated that rapid tumor growth and shortened disease-free survival are associated with high Ki-67 expression, which is correlated with involvement of the vascular space [68,73]. Lusby et al. (2013), reported Ki-67 overexpression, with an accompanying loss of ER and PR expression in case of LMS, whereas in metastatic tumors Ki-67 expression with VEGF and survival was higher as compared to the primary foci [74]. D’Angelo et al. (2011), confirmed the fact that high Ki-67 expression correlated with worsened long-term prognosis for the patient (*p* = 0.01). These authors also suggested that simultaneous evaluation of the clinical-pathological markers such as tumor size, mitotic index, and IHC Ki-67, Bcl-2 greatly increases statistical significance (*p* = 0.001) [75]. Recently, Demura et al. (2017), reported lower Ki-67 expression in those patients treated with selective progesterone receptor modulators (SPRMs) whose tumor volume significantly decreased, and claimed it was an antiproliferative and a proapoptotic effect of the treatment [32].

**Figure 1:**
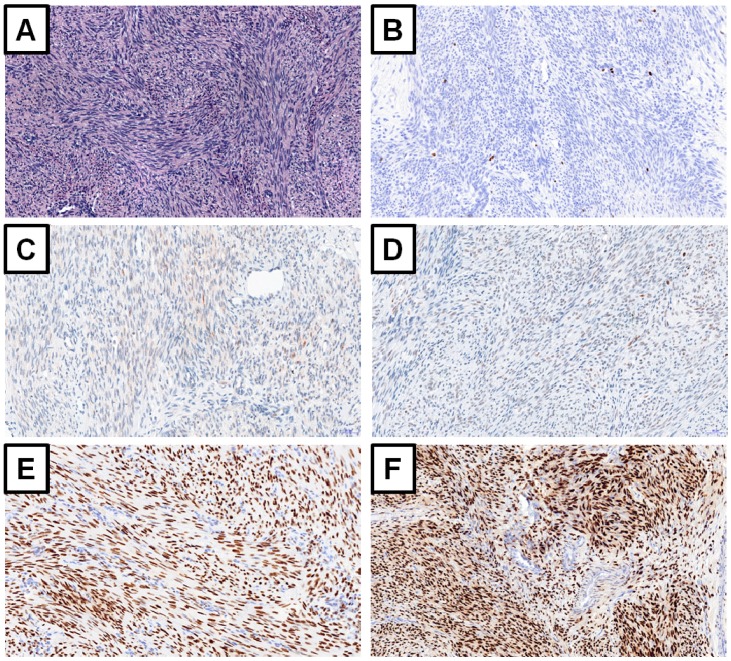
Leiomyoma. (**A**) Topographical staining hematoxylin and eosin. Immunochistochemical expression of (**B**) Ki-67 antigen, (**C**) p53 protein, (**D**) p16 protein, (**E**) estrogen and (**F**) progesteron receptors. Magnification ×20.

In light of the aforementioned data, it seems unquestionable that Ki-67 is a highly useful marker for differentiating between malignant and benign tumors, determining the prognosis for patients with this rare malignancy (LMS), and also planning further oncologic treatment [22]. The diagnostic value of Ki-67 is not to be underestimated; however, certain discrepancies between the reported results explain why Ki-67 has not become a part of the diagnostic panel. A potential panel is presented in Figure 1. 

#### 3.1.2. Tumor Protein p53 (p53, Cellular Tumor Antigen p53)

According to the available data, the *TP53* gene is the most frequently mutated gene in human cancer. The *TP53* gene encodes more than 15 protein isoforms of various sizes. These p53 proteins are known as the p53 isoforms [76]. The p53 protein plays a crucial role in multicellular organisms, where it prevents cancer formation, thus functioning as a tumor suppressor [76]. The p53 protein is engaged in the regulation of numerous cellular processes. It is responsible for the activation of the mechanisms of DNA repair and induction of apoptosis in response to DNA damage, thus being an example of a suppressor protein. Also, p53 displays features of a transcription factor, so mutations within the DNA-binding domain inhibit the transcription of protein-encoding genes, which are responsible for cell protection against tumor invasion [77,78]. Mutations in the *TP53* gene which encodes the p53 protein are correlated with unfavorable prognosis for the affected patients [70].

The analysis of p53 expression has been widely applied in differential diagnosis of various types of tumors, including lesions derived from uterine smooth muscle cells [78]. It seems that the background for using p53 expression in differential diagnosis is strong. The evidence may be found in studies by O’Neill et al. (2007), and Chen et al. (2008), who revealed that expression of p16, p53 and Ki-67 is stronger in uterine LMS as compared to normal LMs, LM variants, and STUMP [31,79]. Dastranj Tabrizi et al. (2015), and Azimpouran et al. (2016), confirmed the previous findings, even for separate p53 evaluation in differential diagnosis of uterine LMS vs. LM and STUMP [80,81]. Zhou et al. (2015), confirmed the overexpression of the p53 protein in uterine LMS. No significant correlations with age, tumor size, clinical stage were found [82]. Layfield et al. (2000), demonstrated an association between p53 expression and the prognosis for patients with LMS [83]. In an earlier study, Blom et al. (1998), found no such link, but they did find a connection between p53 and the frequency of recurrence in LMS [84]. Similar findings were reported by Maltese et al. (2018), who very recently described that ER status (*p* = 0.027) and p53 expression (*p* = 0.015) predicted the risk for relapse in LMS [85]. Notably, p53 positivity in the case of malignant lesions is usually spread, as opposed to focal reaction in the case of benign changes. However, that fact should be treated merely as additional information and not as conclusive evidence [86]. Dall’Asta et al. (2014), recommended using IHC verification of p16 and p53 overexpression in order to identify patients at an increased risk for disease recurrence, who may benefit from aggressive oncological treatment [62]. 

The aforementioned authors suggest that the p53 protein is one of the key elements of differentiating between smooth muscle cell-derived tumors and should be routinely used as a part of the diagnostic panel (Figure 1). The protein in question might not only provide better differentiation but also help to determine the malignancy potential and further prognosis for LMS patients. 

#### 3.1.3. p16 Protein (Cyclin-Dependent Kinase Inhibitor 2A, Multiple Tumor Suppressor 1)

The p16 tumor suppressor protein, encoded by the *CDKN2A* gene [87], plays a crucial role in cell cycle regulation, especially by decelerating cell progression from G1 to S phase [87]. The p16 protein inhibits cell cycle control using the pRB protein [88]. At present, p53 is the second (after pRB) most often measured suppressor protein. The altered expression profile of p53 is found in various tumors, e.g., laryngeal, esophageal, cervical cancers or malignant melanoma [89]. Diagnostic sensitivity is particularly increased when differentiating between cervical intraepithelial neoplasia (CIN)2 vs. CIN3 of cervical cancers associated with HPV (human papilloma virus) infection [90,91]. 

As far as myometrial changes are concerned, most authors report that IHC p16 expression increases with tumor aggressiveness. In 2007, O’Neill et al., analyzed the expression of p16, p53 and Ki-67 in myometrial lesions and found their levels to be elevated in uterine LMS as compared to normal LMs, LM variants, and STUMP [79]. In 2008, Gannon et al., and Chen et al., revealed a statistically stronger expression of p16 in LMS than in LM and its various subtypes (*p* < 0.001) [31,33]. As far as the findings of O’Neill are concerned, Chen et al., also stated that the use of a panel of antibodies to p16, p53, and Ki-67 is very helpful in distinguishing LMS from cellular LM and usual LM [31]. These reports are consistent with the results of Bodner-Adler et al. (2005), who found p16 expression in 12% of LMs, 21% of STUMP, and 57% of LMS cases. The aforementioned authors found statistically significant differences regarding p16 expression in LMS in comparison to STUMP lesions (*p* < 0.05) as well as LMS vs. LMs (*p* < 0.05), whereas the STUMP vs. LM difference was statistically insignificant (*p* > 0.05). Staining intensity differed significantly between LMS and LM and between LMS and STUMP (*p* < 0.05), but no statistically significant difference was observed between STUMP and LM (*p* > 0.05). No statistically significant correlations could be found between p16 expression and clinical stage, age, vascular space involvement, and disease recurrence in patients with LMS (*p* > 0.05). Interestingly, p16 positivity had no differential value (*p* > 0.05) as far as the overall survival is concerned [92]. In 2011, Hakverdi et al., reported p16 overexpression in LMS, suggesting that p16 might be a useful IHC marker in distinguishing uterine LMS from LM and its benign variants [93]. 

More recent studies argue in favor of using IHC panels as markers for the evaluation of uterine LMS, with the loss of ER and PR expression, overexpression of Ki-67, and altered p53, RB, p16 expressions [74]. This is due to fact that in some studies statistical significance was not reached. In a study by Liang et al. (2015), the expression of PR, p16, and phosphorylated histone H3 (pHH3) was found to be significantly different between atypical LMs and LMSs, but there were also no statistically significant differences between atypical LMs and LMs [94]. Finally, Schaefer et al. (2017), analyzed the expression of p16 and p53 on the mRNA and protein level in differential diagnosis between LMS and inflammatory myofibroblastic tumors (IMTs). p16 loss was detected in 5 out of 10 myxoid tumors and 2 out of 11 LMSs, but it was not found in IMTs (*p* = 0.0005), correlating with CDKN2A deletion (*p* = 0.014). Strong p16 staining in 6 out of 21 LMSs and 3 out of 26 IMTs did not correlate with changes in CDKN2A. Schaefer et al., concluded that abnormal staining for p53 and p16 loss was observed more frequently in uterine LMSs, with 100% specificity and 70% sensitivity against IMTs [34].

The aforementioned studies emphasize the importance and the possible role of p16 in differentiating between myometrial changes. In our opinion, the p16 protein should be included in the basic diagnostic panels (Figure 1). 

#### 3.1.4. Proliferating Cell Nuclear Antigen (PCNA)

PCNA is a proliferating cell nuclear antigen—a sliding DNA clamp and an auxiliary protein—which increases polymerase activity approximately 1000-fold, preventing its separation from DNA [95]. PCNA was originally identified as an antigen expressed in the nuclei of cells during the DNA synthesis phase of the cell cycle [96]. It is involved in processes like DNA replication and repair, chromatin remodeling and epigenetics. High PCNA expression in proliferative cells is the reasons why it is considered to be a cell proliferation marker [95]. In a normal healthy tissue of the uterine muscle, both in the secretory and proliferative phases of the cycle, PCNA expression is low, as opposed to a very high expression, especially in the secretory phase, in the pathologically changed tissues of the uterine muscle, e.g., in LMs [18]. According to Vu et al. (1998), a positive PCNA expression is observed in as many as 874/1000 LMS cells, in contrast to 381/1000 in patients after treatment with gonadotropin-releasing hormone (GnRH) agonists, which is evidence for decreased proliferation in response to pharmacological treatment (*p* < 0.001) [97]. 

Also, PCNA allows us to differentiate between malignant and benign lesions derived from smooth muscle cells of the uterus [38]. Higher PCNA expression is observed in LMS as compared to cellular and atypical LM which, together with lowered ER and PR expressions, makes it possible to differentiate between the lesions (*p* < 0.01) [45]. According to Mittal and Demopoulos (2001), and Zhu et al. (2003), IHC using PCNA can be useful in distinguishing between cellular LM and malignant tumors, and can be treated as an IHC marker [45,69]. Guan et al. (2012), reported similar findings when they found the levels of Ki-67 and PCNA expression to be lower in cellular LMs than in LMSs (*p* < 0.05) [38].

An analysis of LM tissues from patients undergoing pharmacological treatment also generated interesting observations about PCNA. Ulipristal acetate (UPA) belongs to a group of SPRMs. UPA has partial agonistic as well as antagonistic effect on the progesterone receptor. It also binds to the glucocorticoid receptor, but has no relevant affinity to the estrogen, androgen and mineralocorticoid receptors [98]. In a study by Yun et al. (2015), who evaluated changes in proliferating and apoptotic markers of UFs after SPRMs or GnRH agonists, PCNA and caspase-3 protein expression was found to be higher in the LM tissue after SPRMs in comparison to the control group (no difference between the control and GnRH agonists groups) [99]. In a study by Luo et al. (2010), who evaluated the effects of different SPRM—telaprisone on proliferation and apoptosis in cultured LM cells, treatment with this drug also significantly decreased the levels of the proliferation marker PCNA and the anti-apoptotic protein Bcl-2 [100]. Epigallocatechin gallate (EGCG) is the ester of epigallocatechin and gallic acid that can be found in black or green tea [101,102]. EGCG has various biological effects confirmed in laboratory studies, and some of them were also performed on LMs. In 2010, Zhang et al., found that cultured LM cells treated with EGCG showed an inhibition of cell proliferation. An extensive further analysis confirmed a significant decrease in the expression of PCNA and Bcl-2 [102].

All these studies on drugs and proliferation markers confirm the effect of the aforementioned substances on the proliferative processes of LM cells. The effect of the pharmacological treatment on the later histopathological differentiation needs further consideration. The literature offers little, if any, data on the matter. Since medications for LM may affect the IHC markers, mistaking malignant lesions for benign pathologies is a risk which should not be ignored. Therefore, it should be obligatory to inform the histopathologist about each case of pharmacotherapy before surgical intervention for LM. Further research about the effects of pharmacotherapy on IHC proliferation markers is necessary. 

PCNA also seems to be a promising IHC marker. In our opinion, and based on the reports in the literature, PCNA improves the detection rates of malignant lesions and may be used as a proliferation marker in cases of smooth muscle cell-derived uterine changes after pharmacological treatment. 

#### 3.1.5. Bcl-2 Protein (B-Cell Lymphoma 2)

Bcl-2, a protein encoded in humans by the *BCL2* gene, plays a major role in the regulation of apoptosis. Damage to the *BCL2* gene has been identified as a causative agent in various cancers where overexpression of the anti-apoptotic genes and under-expression of the pro-apoptotic genes might occur [103,104,105]. Additionally, which is important in the process of carcinogenesis, the Bcl-2 protein may initiate cell replication, thus decreasing the need for growth factors [106].

As far as LM diagnostics and Bcl-2 are concerned, higher Bcl-2 expression was demonstrated in LMs cell as compared to the normal, healthy myometrium [107,108]. In a study by Bodner-Adler et al. (2004), Bcl-2 was expressed more often and more strongly in LMs as compared to LMS and STUMP (Bcl-2 was present in 12 out of 21 LMS, 8 out of 22 STUMP, and 20 out of 25 LMs cases). Statistical significance of Bcl-2 expression was observed between LMS and LMs, and between STUMP and LMs (*p* ≤ 0.05), although no statistical significance has not been found between LMS and STUMP [109]. Zhai et al. (1999), also demonstrated Bcl-2 overexpression in benign uterine smooth muscle tumors (LM, cellular LM, and STUMP) as compared to LMS (*p* < 0.05) [110].

According to the available literature, Bcl-2 and its encoding gene seem to be effective tools to determine the malignancy potential of various tumors and patient prognosis. Banas et al. (2017), recently found that Bcl-2 and selected DNA fragmentation factors are significantly under-expressed in uterine LMS, but only lack of DNA fragmentation factor 40 and Bcl-2 negatively influences disease-free survival and the overall survival [111].

In a study by Conconi et al. (2017), the presence of multiple *BCL2* gene copies and their expression in suspicious STUMPs and relapsed tumors was confirmed. These authors concluded that amplification of the *BCL2* gene present in the STUMPs and its multiple copies suggest its potential role as a marker of STUMP malignancy potential [112]. Smaller involvement of the vascular space and longer survival rates were observed in patients with LMS and positive Bcl-2 expression, which allows us to classify that protein as an effective prognostic marker for patients with LMS [113]. According to Lusby et al., high Bcl-2 expression also predicted longer disease-specific survival in women with uterine LMS [74]. The same observations were made by D’Angelo et al. (2011), who concluded that a combination of clinicopathologic parameters including Ki-67 and Bcl-2 protein expression allows us to distinguish groups of LMS with different survival, and that tumors that were Ki-67 positive and Bcl-2 negative had worse prognosis [75]. On the other hand, in 2016 de Graaff et al. reported that high expression of Bcl-2 proteins might contribute to increased chemoresistance of soft tissue LMS [114].

The abovementioned studies presented evidence that Bcl-2 may be used as yet another marker for differentiating between malignant and benign uterine smooth muscle cell tumors. Regardless of the findings of de Graaff et al. [114], Bcl-2 seems to be a valuable prognostic factor for LMS lesions. More data on the matter might be the key component when planning treatment regime for malignant uterine smooth muscle cell tumors. Further research is necessary as the number of reports in the literature is still rather limited. 

#### 3.1.6. Other Markers

A number of other markers of myometrial differential diagnosis have been described in the literature. Alas, they do not have strong evidence to support their diagnostic value. However, in our opinion, they should be considered in future studies. 

Bodner-Adler et al. (2004), studied matrix metalloproteinase 2 (MMP-2) expression in myometrial lesions and suggested that MMPs play an important role in tumor invasion and metastasis because of their influence on the degradation of the extracellular matrix components. These authors reported stronger MMP-2 expression in patients with LMS as compared to STUMP (*p* = 0.025) and LMs (*p* = 0.006), and concluded that MMP-2 might be a useful IHC parameter to differentiate borderline cases [109]. MMP-2 negative uterine LMSs were also found to have decreased vascular space involvement (*p* = 0.04), and prolonged disease-free survival was observed in MMP-2-negative LMS patients (*p* = 0.09) [115].

In 2006, Bodner-Adler et al. published their results about the expression of thrombospondin 1 (TSP1) in myometrial lesions. According to these authors, TSP1 suppresses angiogenesis by inhibiting endothelial cell proliferation and inducing endothelial cell apoptosis. They observed a stronger expression of TSP1 in LMs as compared to STUMP and LMS (*p* < 0.05), but this significance was not detected between LMS and STUMP [116]. They concluded that a negative correlation between vascular space involvement and TSP1 expression might be a new predictive factor in women with uterine LMS [116].

In 2011, Weissenbacher et al., evaluated the expression of mucin-1, galectin-1 and galectin-3 in tumors derived from the myometrium [42]. Mucins, highly glycosylated proteins, are a component in various secretions [117]. Galectins play an important role in cell adhesion, angiogenesis, metastasis and apoptosis [118]. The expression of Muc-1 was increased in LM and LMS as compared to the normal myometrium. Increased expression of Gal-1 was found in LM in comparison to the healthy myometrium and LMS. The results of Gal-3 expression were statistically insignificant [42].

Very recently, Soltan et al. (2018) published their observations about the expression of galectin-3 and epidermal growth factor receptor (EGFR) in myometrial lesions [39]. EGFR is believed to be one of the most important transmembrane receptors which transduce signals into the cell [119]. In a study by Soltan et al., EGFR overexpression was detected in LMS, while lack of or reduced expression of EGFR was observed in LMs, atypical LMs, and STUMPs. Meanwhile, galectin-3 expression was downregulated in LMS as compared to other myometrial tumors [39]. 

The minichromosome maintenance protein complex (MCM) proteins, which initiate and limit DNA replication, have also been an area of interest for some researchers. According to a study by Chuang et al. (2012), LMs expressed significantly elevated levels of MCM7 as compared to the myometrium [120]. These proteins remain highly stable but active during all phases (G1, S, G2 and M), which is why it might be possible to differentiate between tumor cells during the phases of inactivity and intensive growth [121]. Unfortunately, data on that topic are very limited. 

Major vault protein (MVP)/Catechol-O-methyltransferase (COMT)—Lintel et al. (2018) attempted to assess IHC expression of proteins allowing to distinguish between LM and LMS performed initial proteomic studies to select markers for further evaluation. MVP and COMT had 3.05 and 13.94 times higher expression in LMS relative to LM in ion spectra mass spectrometry, respectively. Subsequently in the IHC, MVP was found to be 50% sensitive and 100% specific when comparing LMS to LM. COMT had a sensitivity of 38% and a specificity of 88%. Immunohistochemical expression of MVP might be suggested as a useful marker in distinguishing LMS from LM in difficult cases [122]. In case of COMT, additional factors may also exert their influence, e.g., unstable concentrations of vit. D (whose influence on COMT has been reported [123]), vit. D supplementation, consumption of large quantities of green tea, or supplementation of its extracts [124].

Cellular retinol-binding protein-1 (CRBP-1) is the carrier protein involved in the transport of retinol from the liver storage site to peripheral tissue [125]. CRBP-1 contributes to the maintenance of the differentiated state of the endometrium through retinol bioavailability regulation. As mentioned by Orlandi et al. (2004), CRBP-1 may help to point the changes which occur in endometrial stroma and therefore be applied as an additional endometrial stromal marker [126]. In 2002, Orlandi et al. found that the expression of CRBP-1 is higher in uterine LMS than in LM and healthy myometrium [127]. In 2007, Zaitseva et al., published their study which demonstrated that CRBP-1 is differentially expressed between myometrium and LM. In this study authors concluded that the expression of CRBP-1 is altered in LMs when compared with healthy myometrium which might be a point for further studies to investigate the importance of these alteration in development of LM [128]. The aforementioned studies suggest that various CRBP-1 expression might represents a new useful marker for the differential diagnosis of smooth muscle tumors of the uterus [127].

Lactate dehydrogenase (LDH) catalyzes the conversion of pyruvate and lactate. It converts pyruvate, the final product of glycolysis, to lactate when oxygen is absent or in short supply, and it performs the reverse reaction during the Cori cycle in the liver. LDH is involved in tumor initiation and metabolism, while cancer cells rely on increased glycolysis resulting in increased lactate production in addition to aerobic respiration even under oxygen-sufficient conditions (in the process called Warburg effect) [129]. Song et al. analyzed the expression and prognostic value of two different subunits of LDH—A and D in LM, cellular LM and LMS. They disclosed significantly stronger IHC expression of LDH-A and LDH-D in patients with LMS vs. LM as well as LMS vs. cellular LM. Moreover, they found that LDH-A-positive LMS patients had a poorer prognosis than LDH-A-negative patients (*p* = 0.03). The authors stated, that overexpressions of LDH-A and LDH-D in LMS patients point to a more aggressive character of the tumor and a positive expression of LDH-A in patients with LMS may have prognostic value in these patients [130].

DNA topoisomerase 2-alpha (TOP2A) is an enzyme that controls and alters the topologic states of DNA during transcription. Baiocchi G et al. (2016) examined protein expression by IHC and gene TOP2A copy number by fluorescence in situ hybridization to verify its prognostic value in malignant (LMSs) and non-malignant (LMs, STUMPs) myometrial lesions; 56.8% of patients with LMS showed high expression of TOP2A. Greater TOP2A levels were found in patients with stage ≥II disease compared with stage I and also in high mitotic index tumors (>20/10 HPF). They stated that protein TOP2A expression does not correlate with TOP2A gene expression and does not predict outcome [131].

Studies about new, alternative IHC markers are necessary. The available diagnostic methods are of high quality but, in many cases, it is still impossible to determine the nature of the tumor and these new IHC markers might be used to improve the detection rates for malignant lesions, which in consequence allows for optimal diagnosis and treatment plan. 

#### 3.1.7. Non-Myometrial Tumors in Differential Diagnosis 

• PEComa—Perivascular Epitheliod Cell Tumor

PEComa is very rare tumor which present co-expression of melanocytic and smooth muscle markers [132]. In a study by Bennett et al., it was clearly shown that all PEComas had expression of HMB-45 antibody, cathepsin K, and at least one muscle marker, with most expressing melan-A (77%) and/or microphthalmia associated transcription factor (MITF) (79%) [133]. What stays in line with the previous observations by Vang et al., with regard to the expression of HMB-45, which is positive for epithelioid mesenchymal tumors of the uterus with an uncertain relationship to pure smooth muscle tumors [134]. Most PEComas can be morphologically distinguished from classical smooth muscle tumors by their distinctive capillary architecture [135]. Future studies about their morphology and IHC patterns for better differential diagnosis are still required.

• Endometrial Stromal Sarcoma (ESS)

In the differential diagnostics of myometrial lesions, in doubtful cases a patomorphologist may also think about neoplasms originating from endometrium, i.e., endometrial stromal tumors. Conklin et al. in 2014 presented new World Health Organization (WHO) classification of endometrial stromal tumors, which recognizes 4 main categories i.e.,: endometrial stromal nodule (ESN), low-grade endometrial stromal sarcoma (LG-ESS), high-grade endometrial stromal sarcoma (HG-ESS), and undifferentiated uterine sarcoma (UUS). These categories are defined by the presence of genetic (distinct translocations) and clinicopathologic (tumor morphology) features. Available genetic diagnostics highlighted the presence of the JAZF1-SUZ12 (formerly JAZF1-JJAZ1) fusion characteristic for ESN and LG-ESSs, the YWHAE-FAM22 translocation identifies HG-ESSs, whereas UUS exhibits no specific molecular pattern [136]. 

For sure, genetic diagnostics are really precise tests (applicable in particular cases) whereas primarily we search for easily available methods of differentiating in the microscopic examination i.e., IHC. There were separate markers tested for their potential usage. Nucci et al. (2001) pointed out that h-Caldesmon appears to be a more sensitive and specific marker of smooth muscle differentiation in the uterus than desmin and may be a useful tool for distinguishing and classifying uterine mesenchymal tumors [137]. This stays in line with study by Rush et al. (2001) [138]. In the same year Chu et al. (2001) analyzed the expression of CD10, desmin, smooth muscle actin (SMA), ER and inhibin in ESS, LM and LMS. They concluded that in combination with SMA, and desmin, CD10 seems to be a useful IHC marker in the differential diagnosis of ESS from LM and LMS [139]. 

Zhu et al. (2004) evaluated the potential utility of a panel of antibodies in the differential diagnosis of ESS and cellular LM. They tested expression of desmin, alpha SMA, calponin h1, h-caldesmon, ER, PR, CD10, CD44v3, PCNA, and mast cells in LG-ESS, HG-ESS, cellular LM and myometrium as well as endometrium. They disclosed that a panel of h-caldesmon, CD10, and CD44v3 might be useful in distinguishing ESS from cellular LM in most cases. Additionally, they postulated a need for further investigation and interpretation of mast cells count as a part of a multivariate approach to the differential diagnosis [140]. 

Moreover, IHC expression of different markers may have prognostic value. Park et al. (2018) evaluated the expression of hormone receptors, i.e., ER, PR, and AR. Their increased expression was associated with significantly better overall survival. When the patients were categorized according to ER, PR, and AR immunoreactivity, triple-positive ESS had the best overall survival, and triple-negative ESS had the worst overall survival. The expression of hormone receptors was associated with favorable survival outcome in ESS. Their predictive value needs further investigation [141].

#### 3.1.8. Steroid Receptors

In light of the fact that the tumors discussed in this article originated from hormonally-active tissue, a short description of steroid receptors seems in order. Specialists who evaluate tumors are obligated to verify the receptor status, but this is not used for differentiation but only for prognostic evaluation (Figure 1). The last decade marked the rise of targeted therapies for various types of neoplastic diseases. The effectiveness of targeted therapies is the result of drug action and patient selection. It is necessary to identify the factor that would supply information about patient response to treatment [142]. As far as uterine smooth muscle cell lesions are concerned, steroid receptors might play such a role. The literature offers numerous publications about studies on ERs and PRs expressions in tissues derived from uterine smooth muscle cells [22,69,72,74,85,94,143]. ER and PR are confirmed in most cases of LMs, and various authors have confirmed different expression of ER and PR receptors in other mesenchymal tumors of the uterine body [13,17,18,85]. The number of publications on the presence of the aforementioned markers in LMS remains limited but, so far, the results presented have been consistent and confirmed decreased ER and PR receptor expression in LMS cells. No differences in the expression of the androgen receptor (AR) in LM and LMS were found (32% vs. 40%, *p* = 0.75) [144]. Azimpouran et al. (2016), reported lack of ER and PR expression in 20 out of 24 and 24 out of 24 patients with LMS, whereas such expression was detected in every case of LM. At the same time, high steroid receptor expression, as was the case with LM, was also confirmed in STUMP changes [81]. These results are consistent with the findings of other authors, who observed a reverse correlation between the ER and PR expression levels and tumor malignancy. Mittal and Demopoulos found a significantly lower number (%) of positive reactions to ER and PR in LMS cells as compared to LM [69]. Similar results were reported by Zhai et al. (1999), who confirmed ER and PR receptor expression in 7% and 36% of LMS cases, respectively [145]. According to other authors, e.g., Lusby et al. (2013), uterine LMS samples exhibited loss of ER and PR expression (with the overexpression of Ki-67 and altered p53, p16 and others) [74]. Liang et al. (2015), demonstrated that high p16 expression and low PR expression suggest the diagnosis of LMS [94]. Additionally, as far as steroid receptors are concerned, ERs can be used to support the gynecologic origin of LMS (3% non-gynecologic vs. 50% gynecologic LMS; *p* < 0.001) [71].

Little is known about the presence of AR in myometrial lesions [27]. Leitao et al. (2004), described positive IHC reactions for AR in 32% of leiomyomas [144]. In a recent study by Baek et al., AR expression was found to be an independent factor for disease-free survival in patients with LMS. No deaths were noted in the AR expression group, and the 5-year overall survival in the AR-negative expression group was 54.8% (*p* = 0.014). Moreover, the co-expression of different steroid receptors (ER and/or PR) with AR was associated with significantly better 5-year disease-free survival and overall survival [146].

In light of the fact that the relationship between these receptors and the development of LMs has been well-documented, it is possible to apply a therapy using selective ER modulators [147,148], GnRH analogs, e.g., leuprolide acetate [99,149], as well as the well-documented therapy using selective PR modulators, e.g., UPA [150,151,152]. It seems prudent to investigate the effects of these drugs on the aforementioned proliferation markers, and in consequence on the histopathological diagnosis. Extensive research is necessary, especially since reports in the literature are scarce.

#### 3.1.9. Future Directions

A vast majority of myometrial lesions leave no room for doubt as far as histopathology diagnosis is concerned. However, there is a need for very thorough differential diagnostics of smooth muscle cell tumors in some patients due to significant clinical and morphological similarities between benign lesions of unknown potential and malignant tumors [22,27,29].

Surgical intervention remains the most common method of treating uterine tumors, especially using minimally invasive techniques [153,154]. The histopathological diagnosis is usually made post-operatively, which might be a cause for concern in case of STUMP, atypical LMs, and LMS. Unfortunately, pre-operative detection rates for these lesions are still very low. Naturally, some changes are suspicious and, in many cases, a seemingly benign lesion might turn out to be lethal. In 2016, Cui and Wright reviewed papers on the prevalence of all uterine cancers in patients who underwent morcellation, and found that the rates of uterine sarcomas in presumed uterine fibroids ranged from 0.00% to 0.49%, and that LMS was the most commonly reported malignancy [155]. Accurate pre-operative diagnosis of LMS was reported in 65% [156] to 84.1% [157] of patients. The topic of undetected LMS was publicized after the 2014 Food and Drug Administration (FDA) warning. The FDA discouraged the use of laparoscopic power morcellation during hysterectomy or myomectomy for LMs due to the risk of dissemination of previously undetected malignancies [158]. The 2017 FDA statement on power morcellation is less radical but still presents the method as potentially risky for the patient and advises caution [159]. Therefore, an accurate and unambiguous diagnosis is important not only due to pre-operative patient eligibility for laparoscopic or classical interventions, but also the subsequent monitoring, therapy and prognosis. Studies on LMS-specific markers which might be used for pre-operative monitoring are practically non-existent, although cytokines, e.g., TNF-α, seem to be a hopeful direction [60].

In doubtful cases, especially differentiating between LM with high proliferative index and LMS, when data obtained from the morphological studies supported by IHC seem to be insufficient or inconclusive, we may also consider molecular analysis. Shikeeva AA et al., investigated the loss of heterozygosity and microsatellite instability to find out genetic differences between the aforementioned types of uterine lesions. In comparative analysis, they disclosed that patients with LMS had much higher frequencies of genetic changes than those with benign tumors. Specificity and sensitivity of the loss of heterozygosity and/or microsatellite instability markers were 92% and 95%, respectively [160].

#### 3.1.10. Summary

Smooth muscle cell tumors belong to the most common lesions of the genital tract [1,2]. Due to their high prevalence, but also a multitude of histopathological subtypes, it is necessary to use molecular techniques in the diagnostic process of suspicious cases. Markers of cell proliferation such as Ki-67 [31], p53 protein [76], and ER and PR expression [144] have been successfully used in differential diagnosis for years. LMs are characterized by low expression of Ki-67 [68,69,70,74,145], p53 [31,74,80,81], and high expression of ER and PR receptors [69,74,145]. It is also a well-known fact that the risk for malignant transformation increases with higher expression of proliferation markers [145,161].

The available markers are undeniably good but some data are missing, and more evidence is necessary to better differentiate smooth muscle cell tumors. In light of that, it is reasonable, if not necessary, to search for new IHC markers of higher sensitivity and specificity [22,27,39,74,82]. Identification of new proliferation markers might herald a breakthrough in tumor differentiation and allow for a more individually tailored approach to therapy planning.

## 4. Conclusions

IHC is easily applicable due to the methodology of obtaining the material for examination (which is similar to routine HE staining). However, it still has its weak sides. Nowadays, in many doubtful cases it is replaced, or better, supported by molecular methods (in first line by: Western Blot, polymerase chain reaction (PCR), enzyme-linked immunosorbent assay (ELISA) or others) as well genetic methods (e.g., evaluation of heterozygosity or microsatellite instability analysis). These are in some cases techniques irreplaceable in differential diagnostics of LMS and proliferative LM of the uterus.

Based on our present knowledge about the applicability of IHC markers in differential diagnosis of lesions originating from the myometrium, we propose focusing on the aforementioned markers, especially gicen that so far, we have not been able to discover unambiguous markers. The use of panels of antibodies is strongly recommended, especially if we bear in mind tumor heterogeneity and semi-quantitative character of this laboratory method. As was clearly demonstrated, markers with strongest differential value are those responsible for proliferation and apoptosis balance. In accordance with global trends, the use of other, new techniques might offer a chance to increase sensitivity and specificity. However, they would also need to be easily applicable, which might present a challenge. 

## Figures and Tables

**Table 1:** The 2014 classification of smooth muscle tumors according to the World Health Organization (WHO) [51].

**Myometrial Neoplasms**	
**Benign Lesions**	
***leiomyoma***	***leiomyoma with Unusual Presentation***
cellular leiomyoma	diffuse leiomyomatosis
epithelioid leiomyoma	intravenous leiomyomatosis
myxoid leiomyoma	benign metastasizing leiomyoma
atypical leiomyoma	
lipoleiomyoma	
**Uncertain Potential**	
***Smooth Muscle Tumor of Uncertain Malignant Potential (STUMP)***	
**Malignant**	
***leiomyosarcoma***	
epithelioid variant	
myxoid variant	

**Table 2:** Differentiation criteria for uterine smooth muscle tumors [53,54].

Benign	Malignant
Low mitotic index (<5 mitotic figures for 10 High Power Field) No cell atypia No tumor cell necrosis (with the exception of ischemic necrosis) Typical presentation of the smooth muscle cells, with uniform shape and size No intravascular component Well-demarcated	Numerous mitotic figures (≥5 for 10 High-Power Field) Significant cell atypia Areas of tumor cell necrosis with island-like presentation

**Table 3:** Leiomyosarcoma of Deep Soft Tissue Stanford Medicine Surgical Pathology Criteria [55].

Leiomyoma *(requires all below)*	Smooth Muscle Tumor of Uncertain Malignant Potential *(used for any of below)*	Leiomyosarcoma *(requires any one of below)*
Cytologically bland	Bland but 1-4 mitotic figures/50 High-Power Field	Cytologic pleomorphism or atypia
<1 mitotic figure/50 High-Power Field	Multiple recurrences but lacking other atypical features	>4 mitotic figures/50 High-Power Field
No tumor cell necrosis		Coagulative tumor cell necrosis

## Abbreviations

AR	androgen receptor
Bcl-2	B-cell lymphoma 2
COMT	catechol-O-methyltransferase
EGCG	epigallocatechin gallate
EGFR	epidermal growth factor receptor
ER	estrogen receptor
ESN	endometrial stromal nodule
ESS	endometrial stromal sarcoma
GnRH	gonadotropin-releasing hormone
HE	hematoxylin and eosin staining
HG-ESS	high-grade endometrial stromal sarcoma
IGF	insulin-like growth factor
IHC	immunohistochemistry
LDH	lactate dehydrogenase
LG-ESS	low-grade endometrial stromal sarcoma
LM	leiomyoma
LMS	leiomyosarcoma
MCM	minichromosome maintenance protein
MITF	microphthalmia associated transcription factor
MMP	matrix metalloproteinase
MVP	major vault protein
PCNA	proliferating cell nuclear antigen
PEComa	perivascular epithelioid cell tumor
PR	progesterone receptor
SMA	smooth muscle actin
SPRM	selective progesterone receptor modulator
STUMP	smooth muscle tumor of uncertain malignant potential
TGF	transforming growth factor
TNF-α	tumor necrosis factor alpha
TOP-2A	DNA topoisomerase 2-alpha
TSP1	thrombospondin 1
UPA	ulipristal acetate
WHO	World Health Organization
VEGF	vascular endothelial growth factor
